# Physician activism in American politics: The opposition to the Price nomination

**DOI:** 10.1371/journal.pone.0215802

**Published:** 2019-06-10

**Authors:** Adam Bonica, Howard Rosenthal, David J. Rothman

**Affiliations:** 1 Department of Political Science, Stanford University, Stanford, California, United States of America; 2 Politics Department, New York University, New York City, New York, United States of America; 3 Center for the Study of Science and Medicine, Columbia College of Physicians and Surgeons, Columbia University, New York City, United States of America; Georgia State University, UNITED STATES

## Abstract

Although a substantial literature considers physician advocacy fundamental to medical professionalism, only a minority of physicians actually pursue it. We analyze the characteristics of 6,402 physicians who engaged in political advocacy by signing the Clinician Action Network's 2016 petition objecting to the American Medical Association's endorsement of the nomination of Tom Price as Secretary of Health and Human Services. These physicians were matched to the NPI (all physicians) and PECOS (largely Medicare payment recipients) directories. Physicians in the directories were matched to publicly disclosed campaign contributions. Contributions are used to measure political preferences expressed on a liberal-conservative scale. We document a pronounced generational realignment in the politics of the medical profession, with recent graduates trending sharply Democratic. Petition signing vs. non-signing is responsive to gender, specialty, geographic location, personal liberal-conservative preferences and year of graduation from medical school. Petition signers were more likely to be women (62% of signers versus 34% of non-signers), recent medical school graduates (58% of signers versus 42% of non-signers), and in lower-paying specialties (27% of signers versus 12% of non-signers). The changing face of physician advocacy has important implications for understanding how the medical profession is likely to influence health care policy in coming decades.

## Introduction

Recent research has emphasized that physicians have become polarized in their political preferences as reflected in their party registrations and contributions [1], [2], [3]. Hersh and Goldenberg [4] have shown that physician’s political beliefs inform their professional decisions regarding patient care. In contrast, we know surprisingly little about physicians who conduct political advocacy, who they are by age, sex, specialty, employment, and geography. Survey research indicates that most physicians consider advocacy part of their professional duties [5]. However, only approximately one-quarter of them pursue the activity [5]. To better understand the characteristics of physicians who do engage in advocacy and to assess its future prospects, we studied the 6,401 physicians who in 2016 signed a public petition objecting to the nomination of Tom Price for Secretary of Health and Human Services (HHS). To be sure, signing a petition is not as demanding as testifying before government committees or helping organize a grass roots movement. However, taking such a stand demonstrates a readiness to express and act on a political position.

Entitled “The AMA Does Not Speak for Us” and sent to both the American Medical Association and members of the U.S. Senate, the petition issued by the Clinical Action Network identified the signers as specialists and generalists whose patients were “the poor and the rich, the young and the elderly” [6]. It declared that by quickly endorsing Price, the AMA had reneged on the physicians’ obligation to “protect and advance care for our patients.” It went on to protest Price’s proposed policies that would dismantle Medicaid, reduce funding for the Children’s Health Insurance Program (CHIP), and privatize Medicare. It concluded by declaring that “the AMA does not represent us, and that we do not subscribe to Dr. Price’s views.” The petition captured media attention, including accounts in the *New York Times*, the *Huffington Post*, and *USA Today* [7], [8], [9].

Price, after a career in orthopedic surgery, entered the House of Representatives in 2005, representing a suburban Atlanta, Georgia district. Of the 441 House members serving in the 114^th^ (2015–16) Congress, Price was the 40^th^ most conservative, firmly among the farthest right members of the Republican party. Conservatism. (The rank is based on DW-NOMINATE scores [10]. We obtain similar results using campaign contribution patterns.) Price’s nomination was contested in the Senate but was approved by a strict party-line vote of 52–47. Price resigned on September 29, 2017 after a scandal involving his use of private jets.

The AMA’s endorsement of Price was consistent with its previous activity as a non-partisan interest group. It contributed to incumbents of both major parties; its two largest donations in 2016 went to Republican representative Paul Ryan and Democratic senator Russ Feingold. The AMA was the 6^th^ largest, by expenditure, lobbyist in 2016. Its campaign contributions dwarfed, by an order of magnitude, those of any other organization of health care professionals. (Contribuions data from www.opensecrets.org, accessed on January 13. 2018.).

## Physicians’ political party alignments

**Fig 1:**
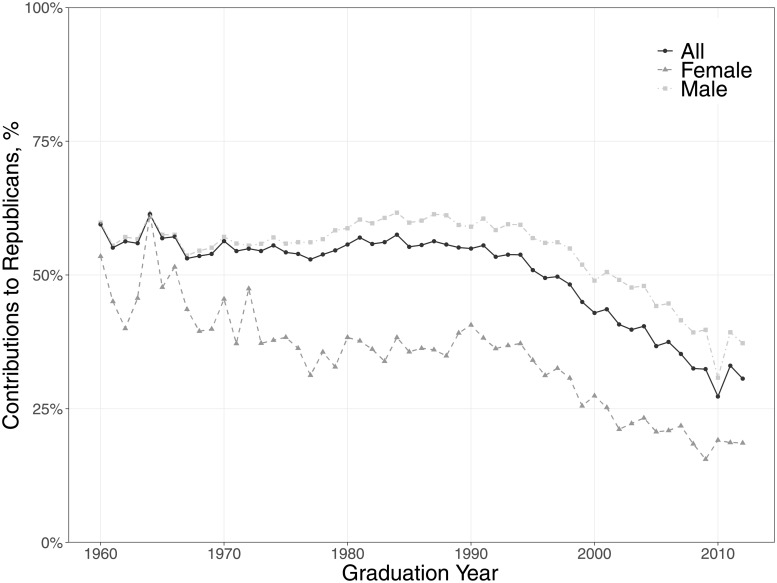
Percentage of campaign contributions to Republicans by year of medical school graduation and gender. Source: NPPES Downloadable File and DIME. Authors’ calculations.

Over the past three decades, data on contributions to presidential and congressional elections indicate that physician party alignment has shifted from a large majority supporting Republican candidates to two sharply divided blocs, with a small majority supporting Democrats [1]. The partisan divisions strongly correlate with sex and specialty; women in lower paying specialties—such as internal medicine, pediatrics, and psychiatry—are far more likely to contribute to Democratic candidates. So too are physicians working in not-for-profit organizations. It is also noteworthy that more recent graduates of medical schools tend to make campaign contributions to Democrats (see Fig 1); that is, younger physicians are much more likely to be Democrats than older ones.

**Fig 2:**
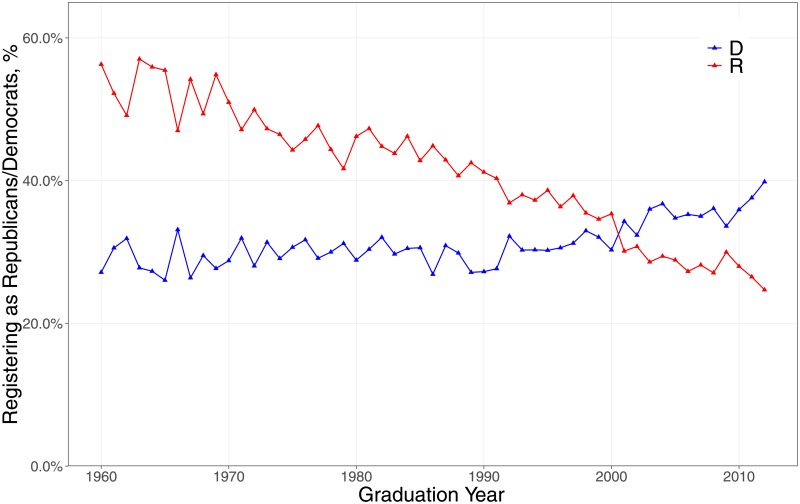
Party registration of Florida physicians by year of medical school graduation. Source: NPPES Downloadable File, Florida Secretary of State, and DIME.

This finding from campaign contributions is confirmed by party registration data from Florida; only Ohio and Florida maintain public electronic records on registration. As Fig 2 shows, physicians who graduated in 1960 and are currently practicing and registered to vote in Florida are much more likely to be Republicans (58%) than Democrats (24%). Physicians who graduated in 2012 are much more likely to be Democrats (41%) than Republicans (24%). Although the percentage of independents rose from 18% to 35% between the 1960 cohort and the 2012 cohort, the net shift to the Democrats of younger physicians is striking. As we shall see, these distinctions mark the characteristics of physicians who joined the anti-Price protest.

## Methods

We matched the 6,401 signatories to the NPI (National Provider Information, downloadable from NPPES, the National Plan and Provider Enumeration System) and PECOS (Medicare Provider Enrollment, Chain, and Ownership System) databases. They were further matched to campaign contribution records from the Database on Ideology, Money in Politics, and Elections (DIME), which combines and standardizes data from the Federal Election Commission and state reporting agencies [11]. Both the NPI and PECOS provide information about the sex, employment, specialty, and geographic location of the physician [1]. PECOS also provides information about the year the physician graduated from medical school, which can be used to study cohort effects. PECOS covers about two-thirds of the physicians in NPI. The difference is mainly one of PECOS not including physicians not receiving Medicare payments. Although PECOS underrepresents pediatricians and some other specialties, the relationship of political behavior to sex, employment, specialty, and geographic location is highly similar when comparing NPI and PECOS.

**Fig 3:**
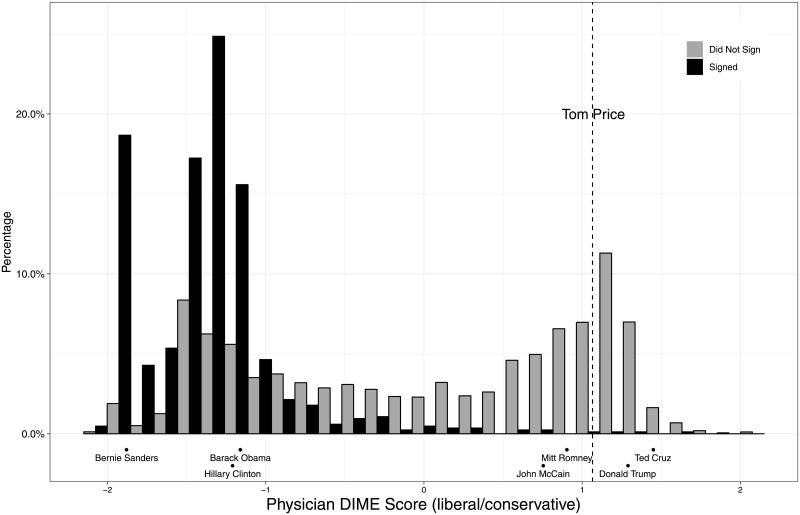
Ideological distributions of physicians. Source: Authors calculations. Data on candidates and physician DIME score are from DIME (Bonica 2016). Note: To aid in interpreting the scale, ideal points for several well-known presidential candidates are shown at the bottom of the figure.

DIME allows us to identify the partisanship of the physician, expressed as the percentage of total contributions to Republicans of contributions to the two major parties. Almost all donors give over 95% of their contributions to one party [12]. DIME also contains a more fine-grained measure of the political ideology of the physician, the DIME score [12]. DIME scores are calculated using a statistical algorithm that analyzes patterns of who gives to whom. Negative scores indicate a liberal ideology, positive ones a conservative ideology. Among Democratic donors, those with DIME scores that are large in magnitude are particularly liberal. The DIME scores allow us to distinguish the liberalism of petition signers among donors. Fig 3 shows the DIME scores of Tom Price and those of recent presidential candidates.

The data we have assembled allows us to compare petition signers to the many non-signers in the NPI or PECOS directories and to compare physician signers who were contributors to physician non-signers who were contributors. The total number of physician records we used from the NPI was 1,044,460, from PECOS, 593,758. The total number of physician contributors in NPI was 311,513, in PECOS, 190,860. Of the 6,401 physician signers, 2,067 were matched to contribution records.

## Results

### Signers of the clinician action network letter

The petition signers were overwhelmingly younger, more female, more likely to work in non-profits, more in low-paying specialties, and more in blue voting areas than the general population of physicians in 2016. They were also more likely to make campaign contributions than the general population of physicians.

Physicians who both donated and signed the petition had characteristics that were especially “blue.” They were far more likely (95%) to have given to Democrats than were those donors who did not sign (49%). They were more female (62% versus 34%) and more likely to work in non-profits (46% versus 32%). They also differed with respect to chosen specialty. Only 4.3% of signers were surgeons versus 9.3% of non-signers. Using Physician Salary Survey Profiles. (Accessed July 26, 2013 at http://www.profilesdatabase.com/resources/2011-2012-physician-salary-survey.) data on earnings by specialty after 6 years of practice, we identified higher paying specialties as those with median incomes above $300,000. Only 13.8% of petition signers were in higher paying specialties versus 32.3% of physicians who did not sign.

NPI doctors who signed were more likely (21%) to have made political contributions in 2015–2016 than those that did not sign (6.3%). The corresponding rates for having donated at any point during the physician’s lifetime are 38.5% for signers and 24.0% for those that did not sign. For signers covered by PECOS, 58% graduated from medical school after 1999 as against 42% of non-signers.

To anchor the results in the context of the Price nomination, 99% of the signers had DIME scores to the left of Price’s. In contrast, 23% of non-signers had positions that were more conservative than Price’s. To anchor the results in the context of the 2016 presidential election, 21% of the petition signers who donated gave to Bernie Sanders as against just 1% of all physicians.

The analysis continues reporting logit regressions. All coefficients are standard rather than marginal effects.

The logit regressions reported in Table 1 show that liberal physicians were far more likely to sign than conservative physicians. The dependent variables is signing = 1, not signing = 0. The results are restricted to those physicians in NPI who made a campaign contribution in 2015–16. The very precisely estimated effect for DIME score in Model 1 shows that, without controls, liberals (negative score) were far more likely to have signed than were conservatives. Model 2 drops observations for physicians not found in PECOS. The estimated coefficient for physician DIME score remains almost unchanged, suggesting that the PECOS subsample is generally representative. Model 3 adds controls for sex, year of graduation from medical school, and the average DIME score of all contributors (not just physicians) in the zip3 of the area where the physician is employed. (The zip3 is the first three digits of the standard five-digit zip code used by the United States Postal Service. Example: 152 is the city of Pittsburgh, PA and suburban areas. The zip3 partitions the country into 929 mutually exclusive geographic areas. The median number of contributors that enter an average DIME score for a zip3 is 18,362.) The positive sign on female shows that females were far more likely to sign than males. The negative sign on zip3 DIME score shows that physicians living in liberal areas were far more likely to sign than those living in conservative ones. Finally, the positive sign on Graduation Year shows that recent graduates are more likely to sign than older ones.

**Table 1:** Effect of ideology, sex, location, specialty, cohort: Logit dependent variable, signed the petition.

	Model 1	Model 2	Model 3
(Intercept)	-6.076	-6.002	-90.174
	(0.054)	(0.061)	(715.918)
Physician DIME score	-1.463	-1.457	-1.060
	(0.039)	(0.044)	(0.048)
Female			0.684
			(0.065)
Zip3 DIME score			-0.989
			(0.082)
Graduation Year			0.035
			(0.003)
Specialty FEs	No	No	Yes
AIC	16520.79	11746.89	11091.10
Log Likelihood	-8258.40	-5871.45	-5449.55
Num. obs.	241,236	174,359	174,359

Note: Fixed effects for 92 specialties not shown.

Table 2 indicates how making a political contribution relates to signing the petition. We use contribution in the 2015–16 election cycle, that is, prior to the Price nominations. Here the dependent variable is signed petition = 1, did not sign = 0. The Ns are much larger than in Table 1 because they include all physicians in NPI (or PECOS). In Model 1, we find that contributors are much more likely to be signers than non-contributors. Model 2 shows that the model 1 result is robust to limiting the sample to physicians in PECOS. Model 3 adds controls for sex, average ideology of zip3 code, and cohort. We find that females, younger physicians, and those in more liberal areas are all less likely to contribute. With the controls, the effect of contributing on signing becomes much larger. That is, the controls adjust for the fact that females and recent graduates predominate among signers. Model 3 further adds control for specialty.

**Table 2:** Effects of contributing in 2015–16 election cycle, sex, and cohort: Logit dependent variable, signed the petition.

	Model 1	Model 2	Model 3
(Intercept)	-5.671	-5.634	-99.376
	(0.018)	(0.021)	(255.085)
Contributed	1.361	1.271	1.644
	(0.039)	(0.047)	(0.049)
Female			0.831
			(0.042)
Zip3 DIME score			-1.479
			(0.050)
Graduation Year			0.040
			(0.002)
Specialty FEs	No	No	Yes
AIC	51926.545	36204.144	32897.716
Log Likelihood	-25961.272	-18100.072	-16352.858
Num. obs.	1,001,467	680,518	680,518

Note: Fixed effects for 92 specialties not shown.

As we are interested as to whether signatories may have been motivated to engage in future acts of political participation, we have collected data on physician donations through the year following the circulation of the petition. Overall, signatories were significantly more likely to donate in the months following the petition. When we subset on contributions made during the three months immediately following the circulation of the petition, we find that 3.6% of signers had donated versus just 0.6% of non-signers. When we subset on contributions made within six months of the circulation of the petition, 4.6% of signers had contributed versus just 0.8% of non-signers. Within a year, 5.7% of signers had donated versus just 1.2% of non-signers.

Table 3 models the likelihood of donating within the 3, 6, and 12 month periods following the signing of the petition. In addition to the full set of controls included above, we include an additional control for having contributed in the 2015–2016 election cycle. The table shows that even controlling for contribution before the Price nomination, signers were significantly more likely to donate during the months following the petition.

**Table 3:** Effects of signing petition, donor status, sex, and cohort: Logit dependent variable, contributing within 3, 6, and 12 months of petition.

	Model 1	Model 2	Model 3
(Intercept)	58.188	58.008	59.950
	(2.098)	(1.588)	(1.370)
Signed Petition	1.119	1.125	1.128
	(0.096)	(0.081)	(0.075)
Contributed in 2016	3.115	3.026	2.976
	(0.026)	(0.019)	(0.016)
Graduation Year	-0.032	-0.032	-0.032
	(0.001)	(0.001)	(0.001)
Female	0.099	0.033	-0.007
	(0.031)	(0.024)	(0.021)
Zip3 DIME score	-0.178	-0.100	-0.059
	(0.031)	(0.023)	(0.020)
Specialty FEs	Yes	Yes	Yes
AIC	62056.073	99776.074	128563.049
Log Likelihood	-30931.036	-49791.037	-64184.524
Num. obs.	680,518	680,518	680,518

Note: Fixed effects for 92 specialties not shown.

There are two plausible interpretations of the results in Table 3: (1) the act of signing the petition caused signers to donate at higher rates in the following months, or (2) the Price nomination and AMA endorsement would have caused the same physicians to donate regardless of the petition, with the petition just providing an early signal of opposition. Either way, this does at least suggest that the Price nomination did activate physicians to donate.

## Discussion

The 6,401 physicians who went public in their advocacy to oppose the Price nomination were exceptionally young and female and tended to live in “blue” parts of the country. Those who donated overwhelmingly gave to Democrats, and particularly to liberal Democrats. Hence, it is not surprising that they signed the Clinician Action Network’s objection to Price’s policy preferences on Medicaid, CHIP, and Medicare.

The shared characteristics of the petition signers suggest that they will continue to pursue advocacy in their careers. Few physicians change the partisanship of their contributions with age, although there is some tendency to moderation of both extremes. Although the petition signers are only 0.6% of the more than 1,000,000 physicians in NPI, they are likely to continue to make political contributions in the future. Our logit results indicate that older cohorts generally donate more than younger cohorts. Thus, donations from the signers are likely to increase over time as they gain more resources. Indeed, our results show that sex differences in political contributions are diminished once one controls for age. So the now younger cohorts of female physicians are likely to become more generous contributors in the future. Many younger physicians are constrained now by relatively low income in residency and medical school debt. With time, however, they may contribute at higher rates as their financial circumstances improve.

The liberal activism of recent medical school graduates, both as campaign contributors and as petition signers, suggests that political advocacy by physicians could occupy a still more significant space within medicine. They may also become educators and models for future physicians.

In substantive terms, the petition singled out for concern proposed reductions in Medicaid, CHIP, and Medicare funding. These issues are likely to persist over time, and physician advocates may well move to promote health care as an entitlement.

## Conclusion

The Clinical Action Network petition was motivated by the AMA endorsement of Tom Price. Signers of the petition manifested "activism" not just in signing but also in being more likely, compared to other physicians, to contribute money to political campaigns. The contributions demonstrated that the signers were likely to be politically liberal or progressive. The petition, through its signers, is indicative of political diversity in a profession that was historically conservative. Political tension within the profession has been reflected in the positions taken by the AMA. After endorsing Price, a strong opponent of Obamacare, the AMA later opposed repeal.
